# Diabetes Self-Management and Education of People Living with Diabetes: A Survey in Primary Health Care in Muscat Oman

**DOI:** 10.1371/journal.pone.0057400

**Published:** 2013-02-22

**Authors:** James A. Elliott, Nadia Noor Abdulhadi, Abdullah A. Al-Maniri, Mohammed A. Al-Shafaee, Rolf Wahlström

**Affiliations:** 1 Department of Public Health Sciences, Division of Global Health, Karolinska Institutet, Stockholm, Sweden; 2 Department of Health Affairs, Ministry of Health, Muscat, Oman; 3 Department of Family Medicine and Public Health, College of Medicine and Health Sciences, Sultan Qaboos University, Muscat, Oman; 4 Department of Public Health and Caring Sciences, Family Medicine and Clinical Epidemiology, Uppsala University, Uppsala, Sweden; The University of Auckland, New Zealand

## Abstract

**Background:**

Although the prevalence of type 2 diabetes in Oman is high and rising, information on how people were self-managing their disease has been lacking. The objective of this study was therefore to assess diabetes self-management and education (DSME) among people living with type 2 diabetes in Oman.

**Methods:**

A questionnaire survey was conducted in public primary health care centres in Muscat. Diabetes self-management and education was assessed by asking how patients recognized and responded to hypo- and hyperglycaemia, and if they had developed strategies to maintain stable blood glucose levels. Patients' demographic information, self-treatment behaviours, awareness of potential long-term complications, and attitudes concerning diabetes management were also recorded. Associations between these factors and diabetes self-management and education were analysed.

**Results:**

In total, 309 patients were surveyed. A quarter (26%, n = 83) were unaware how to recognize hypoglycaemia or respond to it (26%, n = 81). Around half (49%, n = 151), could not recognize hyperglycaemia and more than half could not respond to it (60%, n = 184). Twelve percent (n = 37) of the patients did not have any strategies to stabilize their blood glucose levels. Patients with formal education generally had more diabetes self-management and education than those without (p<0.001), as had patients with longer durations of diabetes (p<0.01). Self-monitoring of blood glucose was practiced by 38% (n = 117) of the patients, and insulin was used by 22% (n = 67), of which about one third independently adjusted dosages. Patients were most often aware of complications concerning loss of vision, renal failure and cardiac problems. Many patients desired further health education.

**Conclusions:**

Many patients displayed dangerous diabetes self-management and education knowledge gaps. The findings suggest a need for improving knowledge transfer to people living with diabetes in the Omani clinical setting.

## Introduction

Oman is a Middle Eastern nation of about 3 million people and currently has one of the world's highest diabetes prevelance. The country has experienced a rapid economic and social transformation since the 1970s, which has resulted in greatly improved living standards [1]–[4]. These changes were noted by the United Nations Development Program, which recently deemed Oman the world's most improved country in terms of human development of the past 40 years [5]. Meanwhile an epidemiologic transition has also taken place. World Health Organization (WHO) data on morbidity and mortality due to unhealthy lifestyles shows a clear shift in disease burden from acute communicable diseases to non-communicable diseases, similar to what has already been observed in several developed countries [6], [7]. Omani lifestyles now feature less physical activity and more unhealthy diets high in the consumption of fast food, refined sugar, and saturated fat. These lifestyle changes are contributing factors explaining why diabetes is now the most prevalent non-communicable disease in Oman [8]. In 2010 the adult diabetes prevalence in Oman was estimated to be 13.4%; the 8 th highest in the world [9]. Experts predict diabetes prevalence in Oman to continue to rise further [10].

Culture and religion have a large influence on behaviour and beliefs regarding health issues and nutrition in the Omani context, impacting the dimensions of culture and social structure that in turn affect the expressions, patterns, and practices of care [11]. The social culture of Oman presents unique difficulties for people living with diabetes in a number of ways, such as the sharing of meals with family and neighbours is a highly valued traditional social interaction [12]. Furthermore, visitors are traditionally offered dates and a local sweet *(halwa)* with coffee upon arrival. This habit is a firmly rooted delight that remains a valued symbol of Omani hospitality throughout the country [13]. In Oman, like in other Gulf States, dates are taken frequently during the day, as there is a strong cultural and religious belief about the nutritional and economic value of dates and it is considered as a blessing fruit according to the holy Qur'an [11]. In smaller amounts dates are useful and nutritious, but the high sugar contents make them unsuitable in larger amounts for patients with diabetes [14]. Moreover, the Omani form of halwa is a particularly sweet gelatinous substance made from sugar, eggs, ghee, honey and spices [13].

Living with diabetes in Oman also brings about another context-specific challenge: the hot and humid climate where temperatures range between 38-48 C° during much of the year [12]. High temperatures make exercise outside nearly impossible before sunset. Exposure to such extreme temperature will quickly destroy insulin and the accuracy of any testing materials. There is also evidence that extreme temperatures alter glucose homeostasis, making blood glucose levels more unpredictable [15].

The Ministry of Health is the main health care provider and provides health services free of charge. The health system in Oman has identified diabetes control as a key health programme priority [16]. Primary health care centres (PHCCs), under the Ministry of Health, are the main entry point of care for most patients with diabetes in Oman, though some are referred to secondary or tertiary care facilities. Diabetes care in PHCCs is provided by general practitioners and practice nurses in scheduled diabetes clinic days. Patients can also access dieticians and health educators for support if referred by the general practitioners. Patients with diabetes usually visit a PHCC every three months. Guidelines for diabetes care are available in all PHCCs; however the current guidelines lack sufficient information on self-management and self-monitoring of blood glucose and how the education on these issues should be done [16].

Due to the nature and complexity of type 2 diabetes, comprehensive and integrated care should be made accessible and affordable for the patients so that they can attain high standard of diabetes management [17]. This includes provision of health education with emphasis on self-management and behaviour change such as: adherence to medications; self-monitoring of blood glucose levels; and proper education about nutrition [17]. Improving diabetes self-management and education (DSME) has been shown effective at improving blood glucose control in multiple large scale studies [18]–[22]. Research has conclusively shown that effective health education should be provided with respect to the patients' level of education and variations in their understanding of the illness [23], [24], since patients with diabetes who had limited literacy and lower knowledge about diabetes and self-management had poorer health outcomes [25].

To date there has been limited research on DSME in Oman, even though it is one epicentre of the global diabetes epidemic [26], [27]. The objective of this study was therefore to assess DSME among people living with type 2 diabetes in Oman. To do so a group of patients receiving routine diabetes care were surveyed at PHCCs in Muscat, the capital city of Oman.

## Materials and Methods

A questionnaire survey for patients living with diabetes was administered by nurses, who were trained in survey techniques, in Muscat PHCCs. A survey of patients attending primary care was judged by the research team to be the most accurate, efficient and rapid method to survey DSME within the Omani population. The survey tool was developed by the research team after reviewing studies conducted in other countries [17]–[25], [28], [29]. The research team members are experienced in diabetes research, public health sciences and three of them are medical doctors worked for long periods in diabetes clinics with a large number of patients. The questionnaire was peer-reviewed by six senior Omani family physicians and subsequently modified. The modified questionnaire was tested in pilot interviews with ten patients with type 2 diabetes attending a secondary outpatient diabetes clinic in Muscat that resulted in constructive changes. Information was collected regarding demographics (e.g., age, sex), duration of diabetes, healthcare utilization, DSME, attitudes towards diabetes management, and treatment practices (see survey instrument Appendix S1). DSME score was assessed through five core open-ended questions: recognition of hyperglycaemia, response to hyperglycaemia, recognition of hypoglycaemia, response to hypoglycaemia, and knowledge of strategies that stabilize blood glucose levels (see scoring protocol Appendix S2). The patients were recruited in 20 PHCCs of the total 26 PHCCs in Muscat Region at the time of data collection.

### Sampling

As previous research revealed a limited understanding of diabetes in the general Omani population [27], our sample was predicted to have a mean DSME score of 5.0/10 (SD = 2.0). It was calculated that 246 persons were needed to complete the survey to achieve a representative sample of people living with diabetes in Muscat, at 5% precision and 95% confidence limit. A non-response rate of 25% was expected, necessitating a minimum sample of 328 participants.

Data was collected from April to June 2010. The patients were recruited according to the following inclusion criteria: Omani citizen, 18 years or older; registered with a diagnosis of type 2 diabetes in the electronic patient database of the Ministry of Health. Patients with type 1 or gestational diabetes were excluded. The nurses were asked to approach every patient who happened to be scheduled for an appointment in their diabetes clinic that day who met the inclusion criteria. The appointment lists in the PHCCs included around 15-18 patients with diabetes who were seen by a general practitioner assisted by one diabetes practice nurse. Patients meeting the study criteria were approached as they arrived for their scheduled appointments. The study aims and the right of refusal were explained to all potential participants. Patients who agreed to participate were interviewed in a private room by a nurse associated with the study. Responses were recorded verbatim. After survey completion patients were educated on knowledge gaps they displayed during the interview.

### Scoring of diabetes self-management and education

A ‘DSME score’ for each participant was calculated from the five core questions (Appendix S2). Each core question was scored 2 points for a correct answer, 1 point if partially correct, and zero for an incorrect answer. One point was subtracted from the total score if a response was actively harmful, for example: insulin in response to hypoglycaemia. Two authors acted as evaluators and scored each survey independently. Consensus was reached between evaluators for all responses. The sum of the scores of the five core questions formed the overall DSME score for each participant. The maximum score was 10 (2 points X 5 questions) and the minimum score was 0. Scores of 8-10 were categorized as *good*, 4-7 as *poor*, and less than 4 as *very poor*.

### Self-treatment, self-measurement of blood glucose and potential complication awareness

Patients were asked if they took oral hypoglycaemic agents (OHAs) or insulin. Insulin-users were asked if they ever changed dosages and when they injected relative to meals. Self-measurement of blood glucose (SMBG) was also assessed. Those not practicing SMBG were asked why they did not. All patients were asked if they could name three long-term complications of diabetes. Verbatim responses were classified into groups, for example: ‘*eye disease*’ and ‘*affects the retina*’ were classified as ‘*loss of vision*’. Patients were last asked what support they needed to manage their diabetes and what they perceived their role was as a patient.

### Data analysis

SPSS Version 19 (IBM) was used for data analysis. Statistical significance threshold used was p<0.05. Pearson’s chi-square tests were used to compare binomial categorical variables, ANOVA for categorical variables with two or more categories and continuous variables, bivariate correlations for comparisons between two continuous variables and independent t-tests were utilized for comparing binomial variables with continuous variables.

### Ethical approval

Ethical approval was received from the Research and Ethics Committee of the Ministry of Health of Oman. The study was conducted in accordance with the World Medical Association’s Declaration of Helsinki. Verbal consent was obtained from all participating patients. Verbal consent, as opposed to written consent, was used due to limited literacy among older patients.

## Results

Of the 370 patients approached, 309 patients (84%) agreed to complete the survey. Those surveyed represented approximately 2.5% of the 12,000 people living with diabetes in the Muscat Region known to the Ministry of Health at the time of sampling. Demographic characteristics of participants are shown in Table 1.

**Table 1:** Demographic characteristics of 309 patients with type 2 diabetes.

		n		%
**Sex**				
Woman		184		60
**Age groups**				
27-39 years		41		13
40-49 years		90		29
50-59 years		93		30
60-83 years		85		28
**Highest education level attained**				
None		163		53
Basic		82		27
Secondary		34		11
Post-Secondary		29		9
**Duration of diabetes**				
Less than 3 years		74		24
3-5 Years		72		23
6-10 Years		81		26
11+ Years		80		26
**Smoking status**				
Yes		30		10
**Previous diabetes education**				
Yes		236		76
**Total**		**309**		**100%**

### Recognition and response to hypo/hyperglycaemia

**Figure 1:**
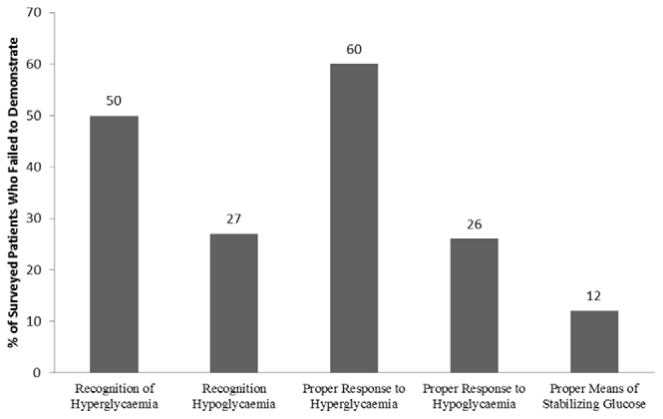
Knowledge gaps among 309 patients, expressed as the percentage of patients with scores of zero or less for the five questions used to develop the DSME score.

More patients could recognise and respond to hypoglycaemia than hyperglycaemia, as seen in Table 2. Over a quarter of the patients (27%; n = 83) were unable to recognize hypoglycaemia or respond to it (26%; n = 81). By comparison, around half of the patients (49%; n = 151) could not recognize hyperglycaemia and 60% (n = 184) could not respond appropriately (Figure 1). In total, 4% (n = 11) of patients gave actively dangerous responses to hypoglycaemia, such as increasing dosages (including OHAs and insulin), or going to sleep. Approximately the same number of patients (3%, n = 10) gave actively dangerous responses to hyperglycaemia, such as drinking juice, or eating sour foods. Most patients mentioned at least one successful strategy for maintaining blood glucose balance (88%; n = 272), for example, exercising or maintaining a healthy diet.

**Table 2:** Diabetes self-management and education scores, treatment practices, previous diabetes education and recognition of complications among 309 patients with type 2 diabetes.

	N		%		Mean DSME Score (Max = 10)
**Sample population**	309		100		5.0
**Diabetes Self-Management and Education (DSME)**					
Good DSME (Total Score: 8-10/10)	40		13		8.6
Poor DSME (Total Score: 4-7/10	191		62		5.5
Very poor DSME (Total Score: <4/10)	78		25		1.8
No recognition of hypoglycaemia	83		27		2.7
Incorrect response to hypoglycaemia	81		26		2.3
No recognition of hyperglycaemia	154		50		4.6
Incorrect response to hyperglycaemia	184		60		4.1
No strategy to stabilize blood glucose	37		12		2.4
**Self-monitoring of blood glucose**					
Yes	115		37		5.9
No	184		60		4.4
Reasons for not practising SMBG					
Not affordable	87		48		4.2
Did not know how	64		34		4.2
Did not want to	50		26		4.9
**Insulin usage**	67		22		5.6
Of insulin users, time injecting relative to meals					
Before meals	49		77		5.9
After meals	10		16		4.6
Sometimes before, sometimes after meals	5		8		5.6
**OHA usage**					
Yes	266		86		5.0
**Previous diabetes education**					
Yes	236		76		5.2
**Listed at least three long-term complications**					
Yes	102		33		6.2
No	201		66		4.4

### Diabetes self-management and education scores

Median and mean DSME scores were both 5.0/10 (range 0-10, SD = 2.3). Seven patients (2.3%) had the maximum score of ten, while eleven patients (3.6%) had the minimum score of zero. DSME scores of the participants are described in Table 2.

**Figure 2:**
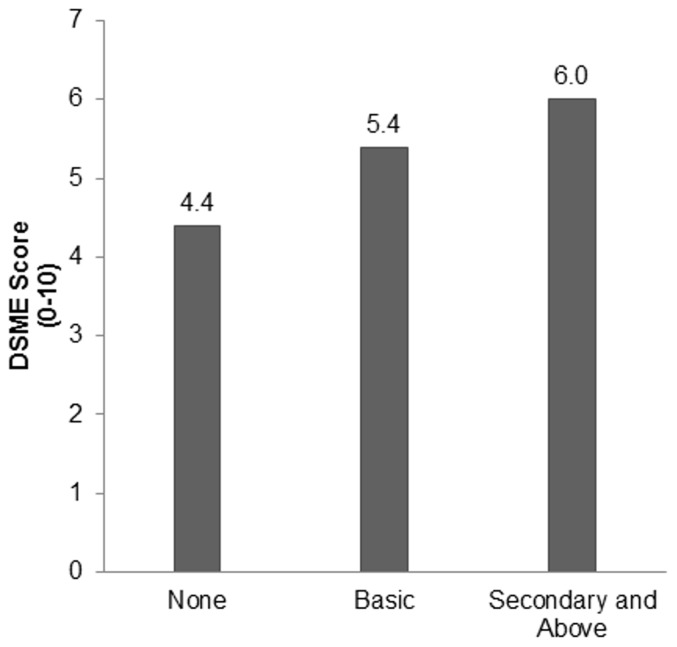
Diabetes self-management and education scores of 309 patients categorized by their level of formal education. A significant relationship was noted (p<0.001).

A significant association (p<0.001) was found between the formal education level of patients and DSME score as displayed in Figure 2. Patients who had completed some formal education were more likely to obtain *good* scores in comparison with those who had not completed any formal education (20% vs. 7%, p<0.001). Patients who reported receiving some form of previous diabetes education also had higher DSME scores (5.2/10 vs. 4.2/10, p = 0.002). Patients who had both formal education and diabetes education were significantly more likely to respond correctly to each of the five core DSME questions (p<0.01), except recognition of hyperglycaemia (p = 0.11). Furthermore, there was a significant positive correlation between DSME scores and duration of diabetes (p<0.01); the longer the duration of diabetes in the subjects, the higher their DSME scores tended to be. There were no statistically significant associations between DSME score and sex, smoking habits, use of OHAs, health care utilization, or past hospitalizations due to diabetes.

### Self-measurement of blood glucose

Less than half of surveyed patients practiced SMBG at any frequency (38%; n = 117). Of those who did, mean frequency was 3.2 times (SD = 3.64) per week (median = 2, range: 1-21). Explanations for not practising SMBG included: costs of glucometers (46%; n = 142); not knowing how (36%; n = 111); and having no desire to do so (26%; n = 80). A significant positive correlation between DSME score and weekly SMBG frequency was noted (r = 0.156, p = 0.006). The difference in DSME score between SMBG practisers and non-practising patients was also significant (5.9/10 vs. 4.4/10, p<0.001).

### OHA and insulin usage

Usage of OHAs (86%; n = 265) was more common than use of insulin (22%; n = 67). One third of patients using insulin self-adjusted their dose if eating smaller or larger meal portions (33%; n = 22) or if experiencing frequent hyperglycaemic or hypoglycaemic reactions (33%; n = 22). Approximately one in five patients using insulin adjusted their dose according to physical activity levels (21%; n = 14). Most insulin users injected before meals (78%; n = 52), though some after meals (13%; n = 9), and others sometimes before, sometimes after (9%; n = 6). Patients using insulin had significantly higher DSME scores (5.6/10 vs. 4.8/10, p = 0.01).

### Recognition of potential complications

A third of patients could name three potential long-term complications of diabetes (33%; n = 103). Most commonly mentioned complications were loss of vision (50%; n = 155), renal problems (44%; n = 136), cardiac problems (20%; n = 63) and foot wound ulcer problems (17%; n = 53). Less frequently mentioned complications were hepatic disorders (6%; n = 19), stroke (5%; n = 16), other vascular and atherosclerotic problems (5%; n = 14), and erectile dysfunction (2%; n = 7). Ability to name three potential long-term complications was significantly associated with higher DSME scores (p<0.001).

### Additional support needed by patients

The most common response by patients when asked what additional support was needed to better manage their diabetes was additional health education (n = 61; 20%). Other responses included additional support from doctors (n = 58; 19%), better medicines (n = 45; 15%), especially long-acting insulin analogs, more affordable SMBG supplies (n = 25; 8%), more support from their family (n = 21; 7%); more support from nurses or dieticians (both n = 19; 6%), and more support from pharmacists (n = 10; 3%).

### Perceived role as a patient

When asked about their role as patients, the most common responses were to be physically active (n = 112; 36%), adherence to medication (n = 64; 21%), to follow the medical advices of health care professionals (n = 23; 7%), self-education about diabetes (n = 16; 5%), to practise SMBG (n = 17; 6%), to attend appointments on time (n = 15; 5%), to maintain a sense of mental wellbeing (n = 8; 3%), and to practise good foot care (n = 7; 2%).

## Discussion

Most patients displayed serious DSME knowledge gaps. One of the most alarming findings was the substantial number of patients who could not mention any signs of abnormal glucose or take corrective measures if detected. A quarter of patients were unable to recognize, or correct, hypoglycaemia. Untreated, hypoglycaemia causes confusion, clumsiness, or fainting, and in severe cases can lead to seizures, coma, and even death. It has been found that frequent episodes of hypoglycaemia eventually stops the release of epinephrine and other stress hormones when blood glucose drops too low, resulting in permanent unawareness of hypoglycaemia [30].

The findings illustrate that greater focus should be placed in the Omani clinical setting on encouraging DSME, making diabetes education accessible, and emphasizing the physiological signs of abnormal blood glucose. Patients should regularly be given opportunities to ask their providers about the causes of abnormal blood glucose and ways in which it can be managed. In particular, patients displaying poor health literacy should always be provided with comprehensive verbal and written information about the complications of diabetes and anti-diabetes medicines, as potentially dangerous hypoglycaemia is a common side effect [30].

The positive correlation between the level of formal education and DSME score found in this study strengthens the body of evidence supporting this link [22]. Limited access to formal education in Oman before the 1970s and limited literacy among people with diabetes may partially explain the major knowledge gaps of the study population, especially in patients over 50 years. In this context, clear, simple and effective communication is essential for the effective delivery of diabetes care. Information given must consider the individual patient's level of understanding and education [31].

Unfortunately, there is often a mismatch between a clinician's level of communication and a patient's level of comprehension. In fact, evidence shows that even in high-income countries like the USA, patients often either misinterpret or do not understand much of the information given to them by clinicians [31]. This lack of understanding, if not corrected by the health care professionals, can lead to medication errors, and adverse medical outcomes.

One of the most positive findings was that nine out of ten patients had developed successful strategies that helped keep their blood glucose stable such as dietary strategies. This may be due to the inclusion of dieticians and health educators in the Omani PHCCs.

Patients who more frequently practised SMBG had higher DSME scores. One plausible explanation is that patients with higher DSME are more motivated to control their disease, resulting in more SMBG. Another is that as patients begin to link SMBG measurements with their symptoms (like exhaustion, thirst) and their ability to recognize abnormal glucose may improve as a result. The findings uncovered barriers to SMBG that can be easily overcome. Slightly more than one third of non-users did not practice SMBG as they simply did not know how. One patient replied in her interview that she thought she was not allowed to buy SMBG devices and assumed they could only be purchased by healthcare professionals.

The percentage of patients on insulin therapy (22%) was found to be slightly lower than in most high-income settings, for instance, 26% in the USA [32]. This may be due to patient factors, such as cultural beliefs about insulin or fear of insulin, or provider factors, such as the belief that insulin is a treatment of last resort [33], [34]. These behaviours can be addressed through health education that emphasizes patient autonomy [35].

Patients with better DSME tended to be more aware of the long-term complications of diabetes. This is an important finding as prevention of complications often depends on the recognition of early disease signs.

The findings of this study highlight the need for appropriate and relevant diabetes education programmes to overcome the knowledge gaps among people living with diabetes in Oman. Many patients expressed a wish for additional health education, suggesting there is a desire for educational programmes that could enhance patient self-management. There is also a lack of knowledge about diabetes in the general population in Oman [27]. The individual's prime role in maintaining health and a satisfying everyday life with chronic conditions like diabetes is increasingly becoming the focus of secondary prevention [36]. Collaborative models of care of self-management education and disease management emphasize empowering patients to find their own solutions. These models speak to a growing realization that people with diabetes are not helped to solve problems or make lasting changes in their lives by simply being told what they should do [37]. In this respect, health professionals' skills to empower patients can be enhanced if they have tools to understand the current motivations and barriers of diabetes self-management of their patients [28]. These crucial approaches can be implemented in Omani health care setting through appropriate planning and strategy by the health care authorities and health policy makers.

The findings of this study emphasize the need to focus efforts on enhancing effective self-management for people living with diabetes in Oman rather than merely conveying information that may not be specific for education on self-management. Moreover, health care providers need to consider the cultural and religious beliefs and values of the patients with diabetes during medical encounters and provision of health education [38].

There are currently no educational facilities to train certified diabetes educators or diabetes specialist nurses in Oman. Increasing the availability of health care professionals with specialist diabetes education training, and increasing the competency and interest in diabetes care among existing providers, would be important steps towards improving DSME [39]. Developing audit and feedback mechanisms that can help better shape clinical organization may be another way of improving diabetes care.

### Limitations

Limitations of this study include those associated with all verbally administered surveys: recall bias, verbal misunderstandings, and the influences of participant and interviewer interaction [40]. The sample size comprised patients solely at the primary care level and did not include secondary or tertiary facilities, patients using private sector health care, health care in neighbouring countries or who were not seeking health care. The study was also conducted in the capital city. Therefore the findings may not directly be applicable to the whole of Oman. However, the structure of primary care is the same throughout Oman, so it plausible that DSME is similar or even lower in other parts of the country due to lower proportion of formal education and other socio-economic factors. The level of formal education in our sample was also quite low, especially in older patients, which calls for caution when generalizing these findings to other settings. Lastly, due to the lack of an established and validated DSME assessment tool in Oman, a newly developed tool was utilized.

## Conclusions

The findings of this study challenge the Omani health system to improve knowledge transfer to people living with diabetes so that they can successfully take on more responsibility for managing their disease. Guidelines need to be further updated and training of providers needs to focus on improving communication skills relevant to knowledge transfer and patient education. It is hoped that the results of this study trigger changes in policy and clinical practice that will translate into better blood glucose control, fewer complications, and a better quality of life for people living with diabetes in Oman and elsewhere. This work can also lead to exploration of DSME in Oman's neighbouring countries and other nations coping with rapid epidemiological transitions.

## Supporting Information

Appendix S1 **Diabetes self-management and education questionnaire.**
(DOC)

Appendix S2 **Diabetes self-management and education assessment scoring.**
(DOC)
