# Supplementary material for: Diabetes Self-Management and Education of People Living with Diabetes: A Survey in Primary Health Care in Muscat Oman
Source: PLoS One. 2013 Feb 22;8(2):e57400. doi: 10.1371/journal.pone.0057400 (PMC3579849; doi:10.1371/journal.pone.0057400)
Supplement: Appendix S1 — Diabetes self-management and education questionnaire. (DOC) [file pone.0057400.s001.doc]

**Omani Diabetes Self-Management Survey**

**First a few questions about you**:

1. Sex: a) Man b) Woman

2. Age:

3. Education Completed: a) None b) Basic education

c) Secondary education d) Post-Secondary education

4. Do you smoke tobacco or shisha? a) Yes b) No

5. How many years have you known that you have diabetes? ______

6. How many times in the past year (365 days) have you been to visit a clinic or any sort of health care facility due to your diabetes?  **______**

7. Have you ever been hospitalized due to your diabetes? a) If yes, how many times?

b) No

8. Have you ever received any form of diabetes education? a) Yes b) No

**Knowledge about diabetes and related practices:**

9. How can you know if your blood sugar is low?

10. What do you do when your blood sugar is low?

11. How can you know if your blood sugar is high?

12. What do you do when your blood sugar is high?

13. What are ways you can help keep your blood sugar from getting too high or low?

14. Can you mention three long-term complications of diabetes?

**Attitudes towards diabetes management**:

15. What kind of support do you need for your diabetes management?

16. In your opinion, please tell me what is your role in your diabetes management?

**Self-management practices**:

17. Do you take insulin(s)?

a) Yes b) No

*IF YES*, please answer the following questions:

i. Do you change your dose if you know you will be eating more or less than usual?

a) Yes b) No

ii. Do you change your dose if you know you will be more physically active than usual?

a) Yes b) No

iii. Do you change your dose if you find your blood sugar is too often high or low?

a) Yes b) No

iv. When do you normally take your insulin with your meal?

a) Before b) After c) Some times before, sometimes after

18. Do you take oral medications for your diabetes?

a) Yes b) No

19. Do you monitor your blood sugar at home? a) Yes b) No

a) If yes, how many times per week: −­­­

b) If no, why not? i) It is unaffordable ii) I do not know how to do it iii) I do not want to

20. Have you gone to an eye-doctor within the last year?

a) Yes b) No
