# Supplementary material for: Diabetes Self-Management and Education of People Living with Diabetes: A Survey in Primary Health Care in Muscat Oman
Source: PLoS One. 2013 Feb 22;8(2):e57400. doi: 10.1371/journal.pone.0057400 (PMC3579849; doi:10.1371/journal.pone.0057400)
Supplement: Appendix S2 — Diabetes self-management and education assessment scoring. (DOC) [file pone.0057400.s002.doc]

**Appendix 2: Diabetes Self-Management and Education Assessment Scoring**

| **Question** | **Appropriate Answers** | **Scoring** |
| --- | --- | --- |
| **How can you tell if your blood sugar is low?** | Cold sweat, shaking, slurred speaking, confusion, lack of coordination, staggering gait, fatigue, nervousness, excess hunger, headache, blurred vision, dizziness, abdominal pain, nausea, SMBG, fainting | *Two Correct = 2*  *One Correct = 1*  *None Correct = 0* |
| **What do you do when your blood sugar is low?** | Consume juice, raw sugar, honey, or any other quick way to ingest sugar orally | *Any Correct = 2*  *None Correct = 0* |
| **How can you tell if your blood sugar is high?** | Thirst, headaches, difficulty concentrating, blurred vision, frequent urination, fatigue, SMBG, persistent vaginal and skin infections, slow-healing wounds, cold or insensitive feet, loss of hair on the lower extremities, erectile dysfunction, chronic constipation, wasting, anorexia, teeth and oral problems, bad breath, palpitations and shortness of breath (ketosis) | *Two Correct = 2*  *One Correct = 1*  *None Correct = 0* |
| **What do you do when your blood sugar is high?** | Drink water, inject insulin, adjust dosage, exercise, and abstain from eating or cut back on portion size | *Two Correct = 2*  *One Correct = 1*  *None Correct = 0* |
| **What are some ways you can help keep your blood sugar from getting too high or low?** | Adjust medication and/or diet, follow an exercise regime, eat consistent food content and amounts, keep regular meal times, test blood sugar regularly, ask for advice from health care providers | *Two Correct = 2*  *One Correct = 1*  *None Correct = 0* |
| *-1 per dangerous response, e.g. insulin for hypoglycaemia response*  **Max Score = 10/10** | | |
